# Eosinophilic Granulomatosis With Polyangiitis (Churg-Strauss Syndrome) Mimicking a Stroke and Acute Coronary Syndrome: A Case Report

**DOI:** 10.7759/cureus.8984

**Published:** 2020-07-03

**Authors:** Uzma Ishaq, Jahanzeb Malik, Adnan Baig, Muhammad Javaid, Umar Ikram

**Affiliations:** 1 Hematology and Medical Oncology, Fauji Foundation Hospital, Rawalpindi, PAK; 2 Cardiology, Rawalpindi Institute of Cardiology, Rawalpindi, PAK

**Keywords:** churg-strauss syndrome, eosinophilic granulomatosis with polyangiitis, eosinophilia, hypereosinophilia, hypereosinophilia syndrome, myocarditis, acute coronary syndrome, rituximab

## Abstract

Churg-Strauss syndrome (CSS) is a rare disease of multiple organ involvement attributed to asthma, eosinophilia, and vasculitis as a diagnostic criterion. Here we report a case of CSS presenting with left leg weakness and chest pain with a diagnosis of myocarditis and neuropathy. Eosinophilia, history of asthma, peripheral neurological damage led to the diagnosis of CSS. Transthoracic echo showed a full-sized segmental wall motion abnormality with normal CT angiography. He responded well to steroid therapy.

## Introduction

Eosinophilic granulomatosis with polyangiitis, also known as Churg-Strauss syndrome (CSS), was first reported in 1951 [1]. It is characterized by a triad of hypereosinophilia, asthma, and necrotizing vasculitis of small to medium arteries in people with a history of atopy [2]. This disease manifests in the third and fourth decade of life affecting both males and females equally [3]. The etiology of CSS is still unknown. It’s likely that a combination of genes and environmental factors, such as allergens or certain medications, triggers an overactive immune system response.

Asthma is the characteristic finding of this syndrome. Lungs are involved frequently with variable involvement of cardiac, vascular, nervous, and renal systems [4]. Cardiovascular system is involved in 30% of CSS cases presenting as pericarditis, pericardial effusion, valvular Loeffler's endocarditis, myocarditis, stroke, heart failure, and myocardial infarction [5]. This is uncommon but cardiac involvement is a major cause of morbidity and mortality in CSS.

Here we report a case of CSS presenting with chest pain and left leg weakness, mimicking acute myocardial infarction, and a stroke.

## Case presentation

A 48-year-old man with a prolonged history of asthma and general fatigue was admitted with three days history of severe, central chest pain radiating to the left arm and jaw. It was relieved by analgesics. He also complained of left-sided lower limb weakness for one day. The patient had no comorbidity.

At presentation, he was accelerated through triage and shifted to coronary care bay for assessment. His blood pressure was 95/60 mmHg with a pulse of 90 beats/min. The chest was clear to auscultation with prolonged expiration and no murmur, rub, or gallop was appreciated on cardiovascular examination. Upper limb power was 5/5 with normal reflexes. In lower limbs, power was 5/5 and 3/5 in right and left leg, respectively. He was suspected of acute coronary syndrome and stroke.

**Figure 1:**
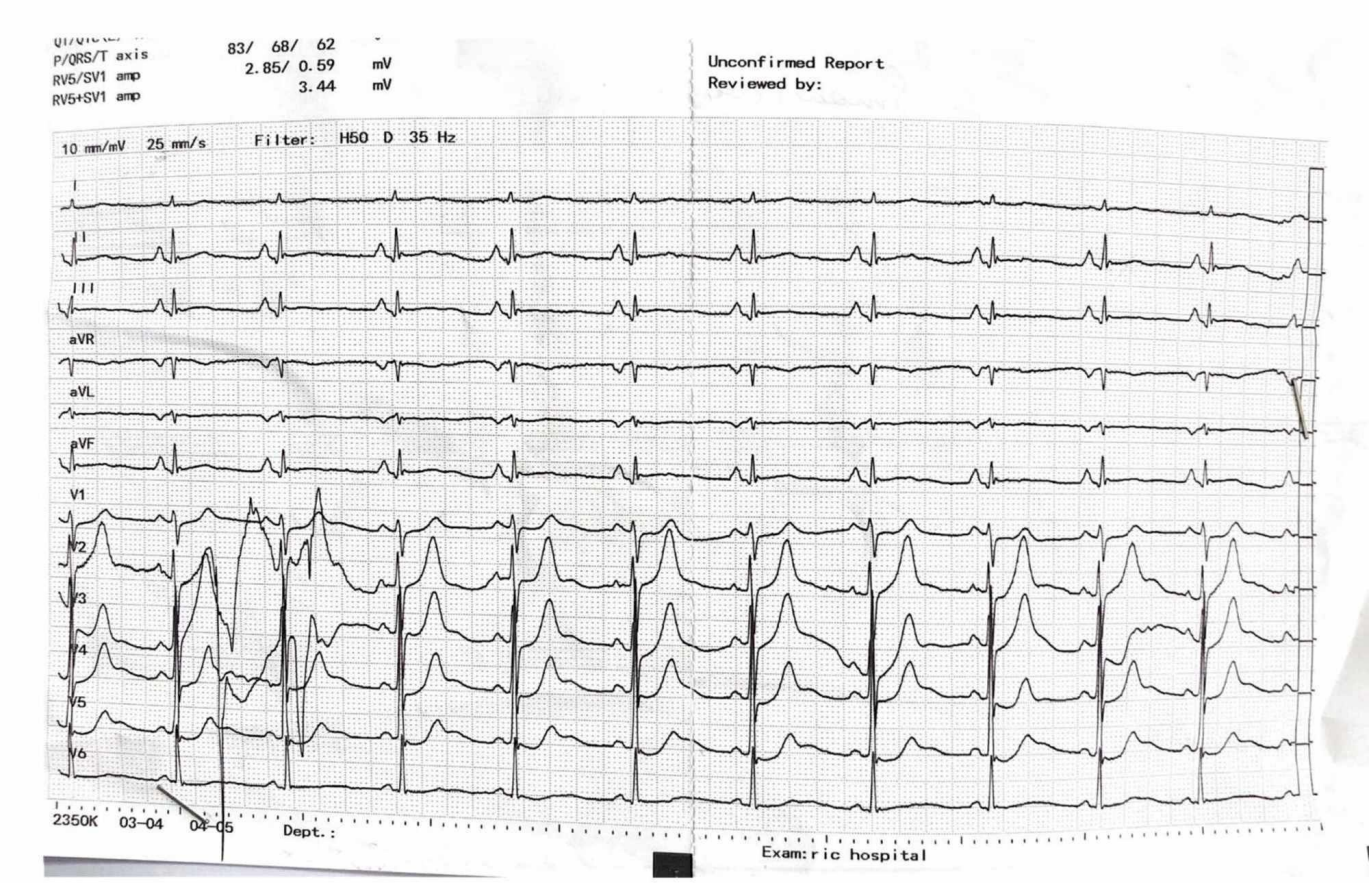
ECG of the patient

ECG was ordered which is shown in Figure 1.

Level of troponin I was 6 ng/mL (normally less than 0.5 ng/mL) and cardiac enzymes were elevated (CK-MB 56 IU/L, CK 252 IU/L). His white blood count was 20 x 10^9^/L and there was eosinophilia with a cell count of 4.5 x 10^9^/L and 56% leukocytes. Hypersensitive C-reactive protein (hsCRP) was 23 mg/dL (normally less than 0.35 mg/dL). Considering his history of asthma, his B-type natriuretic peptide (BNP) was ordered which came out to be 430 pg/mL (normally less than 70 pg/mL) and D-dimer level was 1200 µg/L (normally less than 550 µg/L). Transthoracic echocardiography showed a full-sized and hypokinetic left ventricle with an ejection fraction of 30%. There were severe segmental hypokinesia in the anterior, septal, and apical segments. Echocardiogram for wall motion abnormality is shown in Video 1.

**Video 1:** Wall motion hypokinesia in apical and septal segments

**Figure 2:**
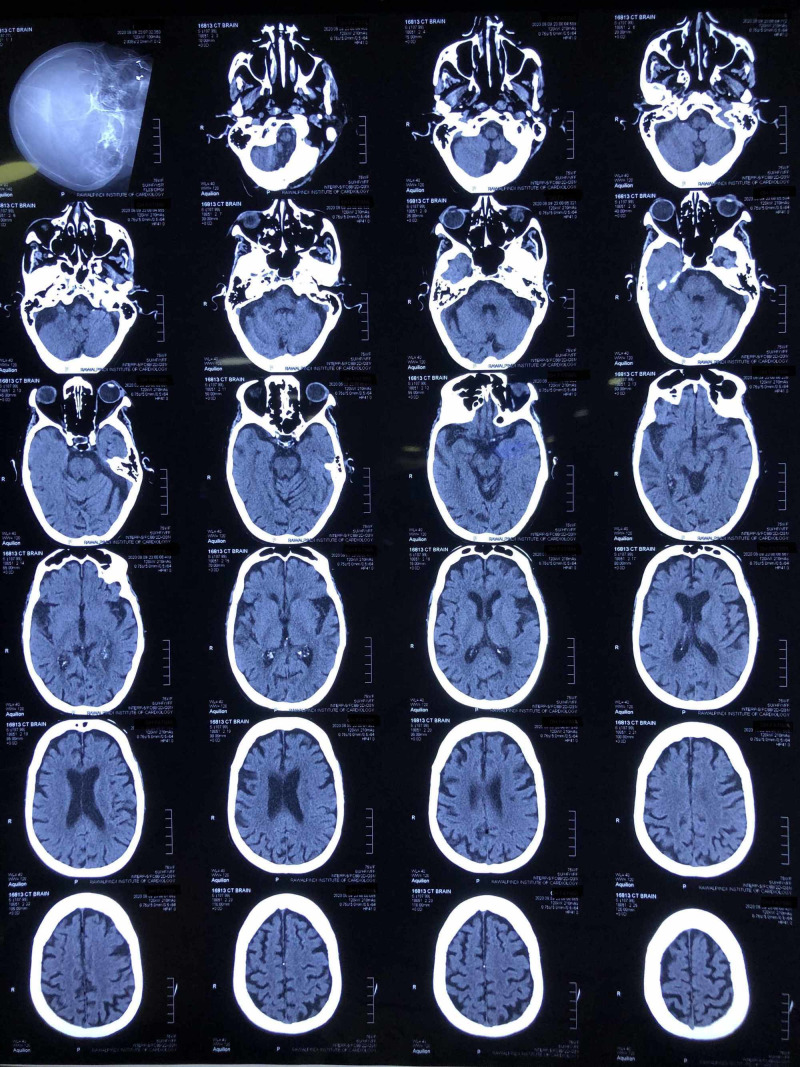
CT scan brain of the patient

CT scan brain is shown in Figure 2 which was normal.

**Figure 3:**
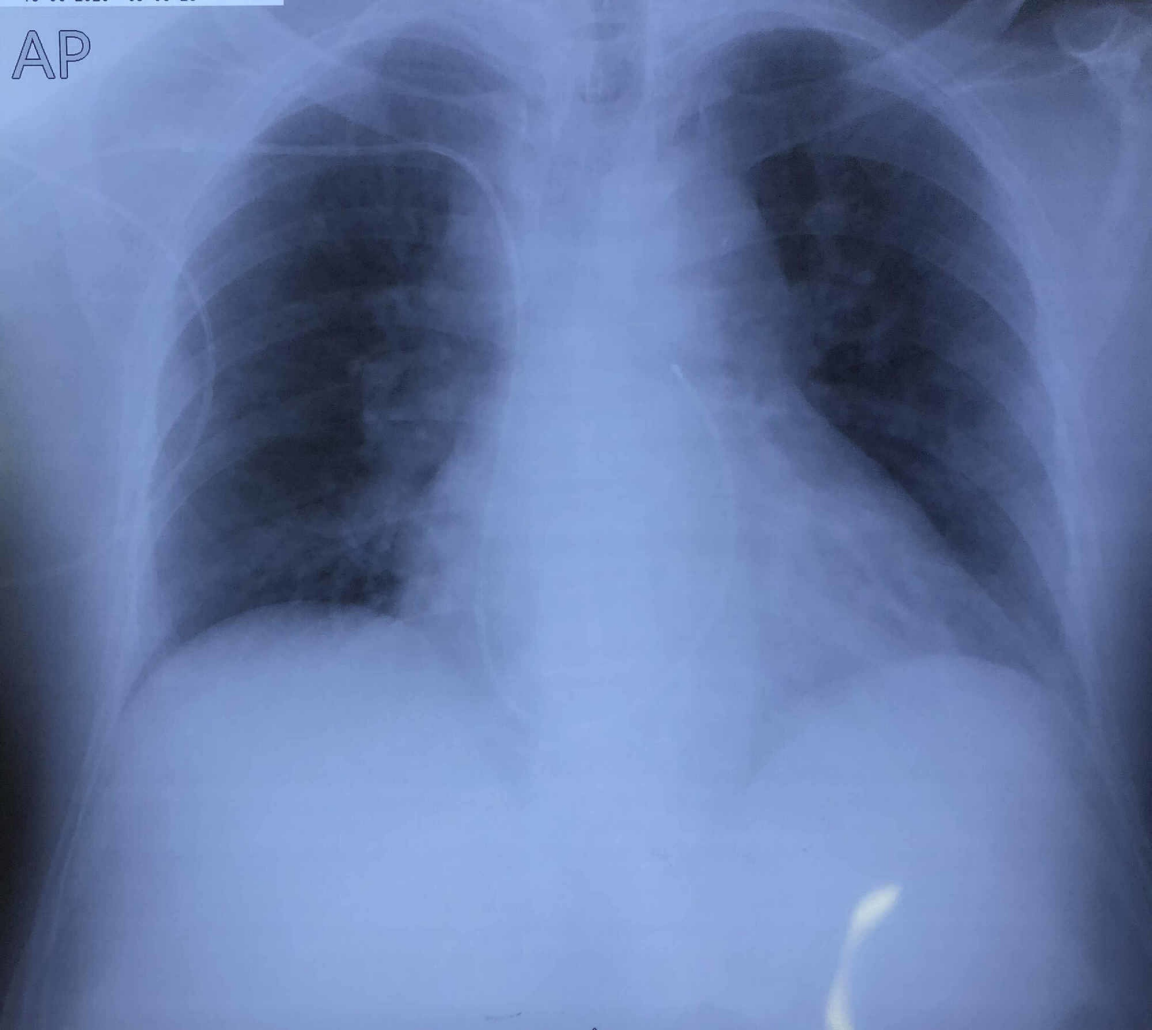
Chest X-ray of the patient

Chest X-ray is shown in Figure 3. It shows no unusual findings.

According to his Global Registry of Acute Coronary Events (GRACE) score of less than 140, he was treated as low risk acute coronary syndrome and his CT angiography was done showing essentially non-obstructive coronary artery disease (Videos 2-4).

**Video 2:** Left anterior descending artery

**Video 3:** Left circumflex artery

**Video 4:** Right coronary artery

Thallium scan revealed multiple perfusion defects in the left ventricle and left ventricle ejection fraction was reduced. On this, a diagnosis of myocarditis was made associated with CSS. Neurology department gave a diagnosis of neuropathy on nerve conduction studies.

Hematology consult was called and his peripheral smear and bone marrow biopsy done which revealed reactive eosinophilia and thrombocytosis. Serum tests were negative for anti-double-stranded DNA (ds-DNA) and anti-neutrophil cytoplasmic antibodies (ANCA). Viral serology was also negative for hepatitis B and C, HIV, and Epstein-Barr virus. The diagnosis of CSS was contemplated given the history of asthma and extravascular eosinophilia. Immunosuppressive therapy was started with prednisolone 1 mg/kg upon diagnosis along with single antiplatelet therapy with aspirin 75 mg once daily. Guide-line directed medical therapy (GDMT) for heart failure was also started. After treatment, his symptoms improved and limb weakness decreased and gradually power became 4/5 at two weeks. His troponin I and hs-CRP levels became normal. Eosinophilia decreased to normal in peripheral smear. The patient was discharged from the hospital on aspirin, ramipril, metoprolol, spironolactone, and prednisolone. One month after diagnosis, the patient was asymptomatic and his repeat echo showed an improved ejection fraction of 50%.

## Discussion

CSS is a rare autoimmune disease with an international incidence of less than 2.5 cases per 100,000 adults per year [6]. It is diagnosed if four principle signs are present: a) asthma, b) hypereosinophilia with an eosinophil count of more than 1.5 x 10^9^/L, c) vasculitis-induced neuropathy, d) migratory or unfixed pulmonary infiltrates, e) paranasal sinus abnormalities, f) extravascular eosinophils on histology and biopsy [7]. Frequently, eosinophilic infiltrates and necrotizing granulomas are also observed [8].

The pathophysiology of CSS is divided into three stages. At first, there is the prodromal stage which is associated with atopy and asthma. It can last for as long as 30 years, according to a report. In the next stage, there is tissue infiltration of eosinophils in the lungs and myocardium. In the final stage, usually, the diagnosis is made and it presents as a vasculitis. Many factors including genes, atopy, drugs, infection, and vaccinations have been implicated with CSS. Dysregulated immune function has been associated with increased eosinophils [9].

Cardiac involvement is very rare but one of the most serious manifestations, accounting for one-half of the deaths associated with CSS [10]. It can be acute, mimicking acute coronary syndrome and it may present in a variety of ways [3]. Myocarditis is another form of cardiac manifestations of CSS, presenting as chest pain, arrhythmias, or cardiogenic shock. Some reports suggest an increased risk of myocarditis in young adults between the ages of 20 to 30 years [11]. There is a marked increase in eosinophil counts in patients with myocarditis, according to some reports [12]. Patients' CRP is increased showing an inflammatory response. However, usually, they are ANCA negative [13].

Immunosuppression therapy remains the mainstay of treatment for CSS and corticosteroids are the first-line therapy, which increases survival [14]. In severe recurrent disease, cyclophosphamide or rituximab can be used [15, 16]. In our patient, he responded well to steroids and remained in remission after a one-month follow-up.

## Conclusions

Being a rare multisystem disease, cardiac manifestations confer increased mortality and morbidity in CSS. Younger females have more incidence of cardiac involvement than older men, and a history of asthma is strongly associated with myocarditis. Markedly increased eosinophil counts are seen in myocarditis than with other organ system involvement and heart disease in CSS is associated with negative ANCA status. As demonstrated by our case, CSS can present as acute coronary syndrome and it can have neurological sequelae.
